# *Salmonella enterica* serovar Paratyphi A-induced immune response in *Caenorhabditis elegans* depends on MAPK pathways and DAF-16

**DOI:** 10.3389/fimmu.2023.1118003

**Published:** 2023-04-12

**Authors:** Ai-Jun Ding, Wei-Ming Zhang, Jian Tao, Bing Chen, Xiao-Cao Liu, Yu Dong, Han-Jing Ma, Shao-Dong Pan, Jiang-Bo He, Wei-Kun Zeng

**Affiliations:** School of Medicine, Kunming University, Kunming, China

**Keywords:** *Salmonella enterica* serovar Paratyphi A, *Caenorhabditis elegans*, MAPK, oxidative stress, immune

## Abstract

*Salmonella enterica* serovar Paratyphi A (*S*. Paratyphi A) is a pathogen that can cause enteric fever. According to the recent epidemic trends of typhoid fever, *S*. Paratyphi A has been the major important causative factor in paratyphoid fever. An effective vaccine for *S*. Paratyphi A has not been developed, which made it a tricky public health concern. Until now, how *S*. Paratyphi A interacts with organisms remain unknown. Here using lifespan assay, we found that *S*. Paratyphi A could infect *Caenorhabditis elegans* (*C. elegans*) at 25°C, and attenuate thermotolerance. The immune response of *C. elegans* was mediated by *tir-1*, *nsy-1*, *sek-1*, *pmk-1*, *mpk-1*, *skn-1*, *daf-2* and *daf-16*, suggesting that *S*. Paratyphi A could regulate the MAPK and insulin pathways. Furthermore, we observed several phenotypical changes when *C. elegans* were fed *S*. Paratyphi A, including an accelerated decline in body movement, reduced the reproductive capacity, shortened spawning cycle, strong preference for OP50, arrested pharyngeal pumping and colonization of the intestinal lumen. The virulence of *S*. Paratyphi A requires living bacteria and is not mediated by secreting toxin. Using hydrogen peroxide analysis and quantitative RT-PCR, we discovered that *S*. Paratyphi A could increase oxidative stress and regulate the immune response in *C*. *elegans*. Our results sheds light on the infection mechanisms of *S*. Paratyphi A and lays a foundation for drugs and vaccine development.

## Introduction

*Salmonella enterica* serovar Paratyphi A (*S*. Paratyphi A) is the causative pathogen of paratyphoid fever and accompanied with fever, abdominal pain, diarrhea, nausea and sometimes vomiting or in extreme cases, it can lead to life-threatening systemic disease (1, 2). In China, the incidence of *S*. Paratyphi A is estimated to range from 0.08 to 192.5 cases for every 100,000 person per year (3). And among the causative pathogen of enteric fever, *S*. Paratyphi A^’^s share ranges from 0.6 to 98.7% (4). The Southeast Asia region presents the third highest incidence of *S*. Paratyphi A-associated infections worldwide, and the incidence of typhoid fever is approximately 110 cases/100 000 population every year (5). Moreover, the occurrence of *S.* Paratyphi A infection has presented a sudden increase in Rourkela, western Odisha (6). A recent report suggested that *S*. Paratyphi A has already spread to countries worldwide (7), which indicates that infection with *S.* Paratyphi A has become a major public health problem. These days, vaccination development for *S.* Paratyphi A focuses on live attenuated vaccines (8, 9), subunit vaccines (10, 11) and conjugate vaccines (12, 13). However, an effective parathyroid vaccine has not been developed. And fluoroquinolones, ciprofloxacin, third generation cephalosporins, nalidixic acid and pefloxacin-resistant *S*. Paratyphi A are gradually increasing (2, 14–17). Therefore, the pathogenic mechanism of *S*. Paratyphi A must be elucidated.

As an invertebrate model, *Caenorhabditis elegans* possess similar host-microbe interactions to the human gut. The virulence and innate immune mechanisms caused by pathogen were reported conserved between *C. elegans* and mammals (18). The human pathogen, such as *Salmonella enterica*, can also infect and lead to pathogenicity in *C. elegans* (19). What’s more, *C. elegans* is known to share conserved defense mechanisms against pathogenic infection (20). Unlike the highly intricate immune network of vertebrates, the original signal network of *C. elegans* make it easier to clarify the relationship among immune signals.

The MAPK pathways, such as PMK-1/p38 MAPK and MPK-1/ERK MAPK, play a major role in defensing against pathogens in *C. elegans*, and MAPK mutants are more sensitive to pathogens (21, 22). Among the immune pathways, Insulin/insulin-like growth factor 1 (IGF-1) signaling (IIS) is conserved in various species. Inhibition of IIS, could induce longevity and improve survival on pathogens (23) and activate DAF-16/FOXO, which is an important transcription factor regulating immune response and stress resistance related genes (24). DAF-16 is not just a target gene of IIS, but also a key molecule to integrate signals from other pathways such as oxidative stress to induce transcriptional changes involved in immunity, stress, and metabolism related genes (25). SKN-1, the other immune-related transcription factor acting the downstream of MAPK pathway and oxidative stress, plays an essential role in detoxification in immunity (26, 27). Despite extensive research on immune pathways, which immune pathways participate in *S*. Paratyphi A infection remains unknown.

Here, we modeled *C. elegans* infection with *S*. Paratyphi A, and found that *S*. Paratyphi A could shorten the lifespan and change thermotolerance and reproductive capacity and spawning cycle and pharyngeal pumping and body movement of *C. elegans*. As secretions and heat-killed treatment of *S*. Paratyphi A exhibited avirulent, *S*. Paratyphi A might infect *C. elegans* by colonizing the intestinal lumen. Hydrogen peroxide assays indicated that *S*. Paratyphi A infection could induce oxidative stress in *C. elegans*. Our results of quantitative RT-PCR indicated that the stress response was changed when worms were fed *S*. Paratyphi A. Genetic analysis with a series of *C. elegans* mutants showed that worms defend against *S*. Paratyphi A via the MAPK pathways and DAF-16. The study elucidated some infection mechanisms of *S*. Paratyphi A and may contribute to the development of drugs and vaccines.

## Materials and methods

### *C. elegans* and bacterial strains

The wild-type N2, PS3551 *hsf-1(sy441) I*, ZD101 *tir-1(qd4) III*, AU3 *nsy-1(ag3) II*, KU4 *sek-1(km4) X*, KU25 *pmk-1(km25) IV*, BJS737 *mpk-1(sbj10) III*, CB1370 *daf-2(e1370) III*, CF1038 *daf-16(mu86)I*, EU1 *skn-1(zu67)IV* and TJ356 zIs356 [*daf-16p*::*daf-16a/b*::*GFP +rol-6(su1006)*] *C. elegans* strains were provided by the *Caenorhabditis* Genetics Center (CGC) and grown at the 25°C (PS3551 *hsf-1(sy441) I* and CB1370 *daf-2(e1370) III* were grown at 20°C) (28). Worms were maintained on NGM plates containing *Escherichia coli* OP50. *E. coli* OP50 and HT115 were procured from CGC, *E. coli* strain JM109 was purchased from Promega Corporation and all grown in Luria-Bertani (LB) medium. Clinical isolates of *Salmonella enterica* serovar Paratyphi A CMCC50973 were provided by the National Center for Medical Culture Collections (Beijing, China) and cultivated in LB broth at 37°C. We constructed the *E. coli*::eGFP and *S.* Paratyphi A::eGFP (both carry the same eGFP expression plasmid pMD18T-*egfp*).

### Lifespan assay

For the wild-type N2 lifespan assay at 25°C, synchronized L4 larvae N2 worms were washed with PBS and then grown on NGM plates containing *S.* Paratyphi A or *E. coli* OP50 and other *E. coli* strains (HT115 and JM109). In the spawning cycle, the nematodes were picked up by wormpicker to new plates every other day as previously described (29).

For the mutant strain lifespan assay, Late L4 larvae N2 worms and mutants were washed with PBS and maintained to NGM plates containing OP50 or *S.* Paratyphi A. Nematodes were picked and transferred to fresh plates with OP50 or *S.* Paratyphi A every other day. Worms were determined as dead if they showed non-response to a mechanical stimulus. Worms were excluded if they leaved plate, appeared swelling of internal organs, or hatched progeny in the maternal worms. The data of lifespan were analyzed by using IBM SPSS version 19.0. Kaplan–Meier lifespan analysis was applied, and log-rank test was carried out to calculate *p* values. The effects of OP50 and *S.* Paratyphi A on the mutant *C. elegans* were analyzed using Cox proportional-hazard models (30). The most obvious results of three independent trials are shown in the curves. Mean lifespan of at least 3 independent experiments are displayed as the mean ± standard deviation. * *p* < 0.05, ** *p* < 0.01, and *** *p* < 0.001 represented three levels of statistical significance when using Student’s *t*-test.

### Thermotolerance assay

To eliminate the effects of reproduction, the thermotolerance assays were performed with hermaphrodites on adult day 5, about the time that egg-laying had finished (31). Briefly, synchronized day 5 worms fed OP50 were washed with PBS and cultivated onto plates with OP50 or *S*. Paratyphi A and incubated at 35°C. Survival was determined every 2 h at 35°C. Kaplan–Meier lifespan analysis was applied, and log-rank test was carried out to calculate *p* values. The most obvious results of three independent trials are shown in the curves. Mean lifespan of at least 3 independent experiments are presented as the mean ± standard deviation. * *p* < 0.05, ** *p* < 0.01, and *** *p* < 0.001 represented three levels of statistical significance when using Student’s *t*-test.

### Body movement assay

Body movement assays were carried out as reported previously (32). To eliminate the influence by thickness of the bacterial lawn on OP50 or *S.* Paratyphi A, and considering that OP50 grow slowly, the assay was performed with bacteria at density of 5x10^9^ and 5x10^10^ bacteria/mL for OP50 group and 5x10^9^ bacteria/mL for *S.* Paratyphi A group. Overnight cultures of OP50 and *S*. Paratyphi A were separately inoculated 1:100 into fresh LB broth. After maintained in a shaker (200 rpm) at 37°C for about 3.5 h, the cultures were stored at 4°C. Serial dilution of cultures was spread on LB plates to determine the bacterial count. According the counts, dilution or concentration by centrifuge were done to adjust the final concentration as OP50 5×10^9^/mL, OP50 5×10^10^/mL and *S*. Paratyphi A 5×10^9^/mL. 200 μL of each bacteria density was added onto 6 cm NGM plates. Then synchronized L4 larvae N2 worms were washed with PBS to remove OP50 and then cultivated to NGM plates containing OP50 or *S.* Paratyphi A. *C. elegans* were at fast moving stages when they crawled continuously in a well-coordinated sinusoidal curve pattern after tapping the plate, otherwise, the worms were defined as a not fast movement. The movement of worms was scored daily. The data of body movement were analyzed by using IBM SPSS version 19.0. Kaplan–Meier analysis was applied, and log-rank test was carried out to calculate *p* values. At least three independent experiments were carried out, and the most obvious result is shown in the curves. Data are presented as the mean ± standard deviation from three independent experiments. * *p* < 0.05, ** *p* < 0.01, and *** *p* < 0.001 represented three levels of statistical significance when using Student’s *t*-test.

### Spawning assay

To analyze the effect of *S*. Paratyphi A on the spawning of *C. elegans*, eggs of N2 worms were grown on plates containing OP50 or *S.* Paratyphi A and incubated at 25°C, and 15 L4 larvae N2 worms were transferred to a new plate every day until eggs laying finished. The number of progenies produced by a specific worm was counted throughout the spawning cycle through everyday interval. The assay was performed in at least three independent trials, and all the results are displayed in this study.

### Chemotaxis assay

To assess food preference in the presence of OP50 or *S.* Paratyphi A on the same plate, chemotaxis assays were performed as previously reported (33). 25 μL of each bacterial suspension was seeded within a circle with diameter of 2 cm on either end of a 60 mm NGM plate, worms were placed to center of the plate where is 1 cm away from the test bacterial spots. Synchronized L4 larvae N2 worms were washed with PBS and trained to NGM plates containing OP50 or *S*. Paratyphi A at 25°C for 12 h. Then, the animals were washed off the training plates in PBS and washed two more times in PBS. Using a wide orifice tip, 20 μL of PBS containing approximately 50-100 nematodes were added to the plate midway between OP50 and *S.* Paratyphi A lawns. The plates were incubated at 25°C for 1 h before analyzing the number of animals on each zone. The chemotaxis index was calculated based on the formula: Chemotaxis index = (No. of worms on OP50 lawn-No. of worms on *S*. Paratyphi A lawn)/Total No. of worms tested. The assay was carried out at least three independent trials, and all the results are displayed in this study.

### Pharyngeal pumping assay

To check the feeding behavior of worms exposed to OP50 and *S*. Paratyphi A, at least 10 synchronized L4 larvae N2 worms were transferred to plates seeded with OP50 or *S.* Paratyphi A and grown at 25°C. The pharyngeal pumping rates of OP50 or *S.* Paratyphi A-exposed nematodes were analyzed at 12 h, 24 h, and 48 h using a stereomicroscope for 20 seconds duration.

### Bacterial colonization assay

The bacterial colonization assay was carried out according to previous reports (34, 35). Age-synchronized L4 larvae N2 worms were washed with PBS and grown on plates seeded with OP50 or *S.* Paratyphi A for 12 h, 24 h, and 48 h at 25°C. Then 10 worms were washed with PBS containing 25 mM levamisole (to stop pharyngeal pumping) and then immersed in 100 μg/mL gentamicin and 25 mM levamisole for 1 h to clear the bacteria on the surface of nematodes and then washed twice more with PBS alone. Then worms were transferred to plates without any food to further remove the antibiotics and bacteria. 10 worms were picked up immediately and disrupted mechanically in 50 μL of PBS containing 1% Triton X-100 in 1.5 mL Eppendorf tube. The worm lysates were diluted in PBS and plated on MacConkey agar at 37°C. After overnight incubation, OP50 and *S.* Paratyphi A colonies were counted and the CFU/worm was calculated.

### Analysis of live bacterial accumulation in intestinal lumen

To confirm that *S.* Paratyphi A could proliferate in the intestinal lumen, synchronized L4 larvae N2 worms grown on OP50 seeded plates were transferred to plates containing *E. coli*::eGFP or *S.* Paratyphi A::eGFP. After ingestion of *E. coli*::eGFP or *S.* Paratyphi A::eGFP for 24 hours, the nematodes were rinsed three more times in PBS to remove residual bacteria and subsequently grown on NGM plates without any bacteria, where they fasted for 24 hours until the fluorescence analysis were carried out (36). The fluorescence microscope system (Life Technologies EVOS FL Auto) was used to detect fluorescence in the intestine under 100-fold magnification.

### Bacterial viability and secretions assay

After overnight growth, *E. coli* OP50 and *S*. Paratyphi A were inactivated at 65°C for 30 min (37). Then the cultures of OP50 and *S*. Paratyphi A were incubated on MacConkey agar at 37°C to check whether any bacteria survived. Synchronized L4 larvae N2 worms were added to plates seeded with heat-killed *E. coli* OP50 or *S*. Paratyphi A, lifespan assay was performed as described above.

To examine whether the secretions of *S*. Paratyphi A affect the viability of *C. elegans*, we carried out secretions assay as previously reported (38). After overnight growth, cultures of OP50 and *S*. Paratyphi A were centrifuged at 5,000 g for 10 min. 0.22 μm syringe filter was used to clear residual bacteria of the supernatant. Heat-killed *E. coli* OP50 were resuspended in the filtered supernatant and added to NGM plates. Then lifespan assay of N2 worms was carried out.

### Subcellular localization of DAF-16/FOXO assay

Synchronized L4 stage of strain TJ356 zIs356 [*daf-16p*::*daf-16a/b*::*GFP +rol-6(su1006)*] were washed with PBS and maintained on plates containing OP50 or *S.* Paratyphi A for 30 min at 25°C. The fluorescence microscope system (Life Technologies EVOS FL Auto) was applied to test the subcellular localization of DAF-16 under 200-fold magnification.

### Hydrogen peroxide assay

Synchronized L4 larvae N2 worms were rinsed with PBS and grown on plates seeded with OP50 or *S.* Paratyphi A for 12, 24 and 48 hours. Worms were washed with PBS and disrupted mechanically. The concentration of protein was quantified with a BCA Protein Assay Kit (Beyotime) for different times. At the same time, the hydrogen peroxide was tested using Hydrogen Peroxide (H_2_O_2_) Content Assay Kit (Solarbio). The data were expressed as μM hydrogen peroxide generation/mg of proteins.

### Quantitative RT-PCR assays

Synchronized L4 larvae N2 worms were collected and washed with PBS to exhaust residual bacteria and then grown on plates with OP50 or *S.* Paratyphi A for 12 h. RNAiso Plus (Takara) was applied to extract total RNA of the samples. At least 2 μg of total RNA was added to synthesize cDNA, which was applied a PrimeScript™ RT reagent Kit with gDNA Eraser (Takara). Quantitative RT-PCR was carried out by applying Power SYBR Green PCR Master Mix (Applied Biosystems) on an ABI StepOne™ system (Applied Biosystems). 2^-ΔΔCT^ method was carried out to calculate the relative mRNA levels of gene tested and *cdc-42* was applied to the reference genes (37). The target gene expression in OP50 group was set as 1 for data normalization, *p* values were analyzed by using two tailed *t* test. The primers designed in this study are displayed in **Supplementary Table 13**.

### Western blotting assay

Synchronized L4 larvae N2 worms washed with PBS to clear residual bacteria were grown on plates with OP50 or *S*. Paratyphi A for 12 h. Worms (KU25 *pmk-1(km25) IV* was analyzed to verify mutant) were rinsed by PBS and then lysed in the RIPA buffer containing protease and phosphatase inhibitor mixture prior to triturating. BCA Protein Assay Kit (Beyotime) was applied to quantify the concentration of protein. After boiled, samples containing 20 μg proteins were separated on 12% SDS–PAGE and electro-transferred onto 0.2 μm polyvinylidene difluoride membranes. After blocked in 5% fat-free milk, The membranes were incubated with primary antibodies against phospho-p38/PMK-1 (1:1000, Cell Signaling) or actin (1:5000, Cell Signaling) followed by horseradish peroxidase-conjugated anti-mouse antibody (1:5000, Thermo Fisher Scientific). Quantification of blot were analyzed using Image J.

## Results

### *S.* Paratyphi A infection affected the phenotype of *C. elegans*

**Figure 1:**
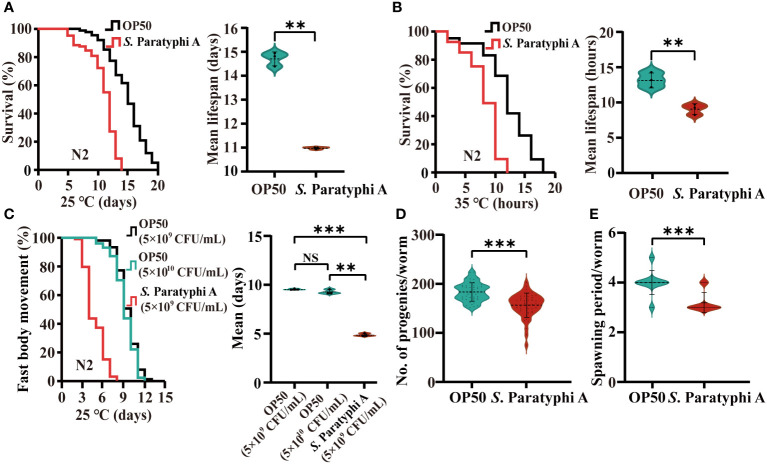
*S.* Paratyphi A reduced the lifespan and thermotolerance, shortened the period of fast body movement and reduced the reproductive capacity of *C elegans*. **(A)** Representative survival curves of N2 of three independent trials and graphs of mean lifespan of at least 3 independent experiments at 25°C, and 35°C **(B)** on NGM plates containing either OP50 or *S.* Paratyphi A. Kaplan–Meier analysis was used to analyze the results of lifespan, and *p* values were calculated based on the log-rank test. Mean lifespan are presented as the mean ± standard deviation. ** *p* < 0.01, Student’s *t*-test. **(C)** Representative curves of three independent trials of age-related movements of worms treated with 5x10^9^ and 5x10^10^ bacteria/mL for OP50 group and 5x10^9^ bacteria/mL for *S*. Paratyphi A group (*p <*0.0001, log-rank test) and graphs of mean fast movement period of at least 3 independent experiments (mean ± SD, ** *p* < 0.01, *** *p* < 0.001, NS represents not significant, Student’s *t*-test). The movements of nematodes were classified as fast and not fast movement. **(D)** Number of eggs laid (mean ± SD, *** *p* < 0.001, Student’s *t*-test) and spawning period **(E)** of worms cultured with OP50 and *S.* Paratyphi A (mean ± SD, *** *p* < 0.001, Student’s *t*-test).

To test the effect of *S.* Paratyphi A infection on lifespan, we explored the lifespan of wild-type N2 hermaphrodites maintained at 25°C on agar plates with *E. coli* OP50 or *S.* Paratyphi A. Compared to OP50, *S.* Paratyphi A infection decreased the average mean lifespan of N2 by about 25% (**Figure 1A**; **Supplementary Table 1**). Compared to other *E. coli* strains (HT115 and JM109), *S*. Paratyphi A also exhibited pathogenicity (**Supplementary Table 2**). Thermotolerance assay also indicated that *S.* Paratyphi A reduced the average mean lifespan of worms by up to 30% at 35°C (**Figure 1B**; **Supplementary Table 1**). The higher temperature than normal is a stress factor by itself. To exclude the factor of heat stress, we also checked the lifespan of mutation of *hsf-1(sy441) I*, which carried the defect of heat shock factor. We found that the lifespan of the *hsf-1(sy441) I* mutant was significantly decreased when nematodes were exposed to *S*. Paratyphi A at 35°C (**Supplementary Table 3**). The mean hazard ratio for *hsf-1(sy441) I* mutant exposed to *S*. Paratyphi A were 6.557 compared to 6.706 of OP50 for the gene (Student’s *t*-test *p*=0.55205). *S.* Paratyphi A infected worms may be independent of stress factor. Worms maintained at higher temperature achieved more distinct lethal effect, suggesting that 35°C was the optimum environment for *S.* Paratyphi A to infect the host. Body movement and reproduction were analyzed to check the physiological status of the worms. We analyzed the body movement of nematodes exposed to 5x10^9^ and 5x10^10^ CFU/mL for OP50 and 5x10^9^ CFU/mL for *S.* Paratyphi A, the bacteria density didn’t lead to dieting (39) and also exclude the effect of thickness of the bacterial lawn caused by the slow growing of OP50. The results showed that 5x10^9^ CFU/mL of *S.* Paratyphi A significantly shortened the period of fast body movement compared to 5x10^9^ and 5x10^10^ CFU/mL of OP50 (**Figure 1C**; **Supplementary Table 4**). Furthermore, the number of eggs laying under the *S.* Paratyphi A treatment was reduced (OP50: 184 ± 19 eggs, *S.* Paratyphi A: 156 ± 25 eggs, *p*<0.0001, **Figure 1D**; **Supplementary Table 5**). And the spawning period of worms was significantly shortened by *S.* Paratyphi A (OP50: 4 ± 0.5 days, *S.* Paratyphi A: 3.2 ± 0.4 days, *p*<0.0001, **Figure 1E**; **Supplementary Table 5**).

### *S.* Paratyphi A-trained worms indicated a strong bias for OP50, showed a decreased rate of pharyngeal pumping, and exhibited increased colonization in the intestinal tract.

**Figure 2:**
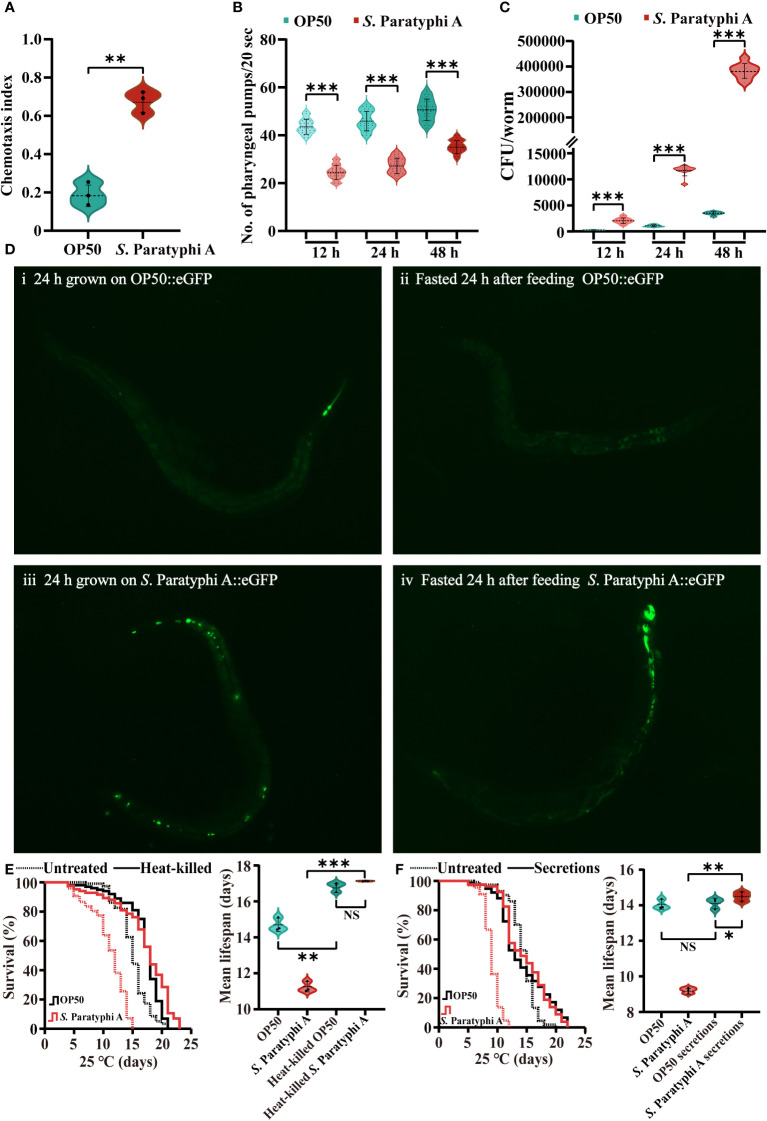
*S.* Paratyphi A-trained worms showed a bias toward OP50, a decreased rate of pharyngeal pumping, and increased colonization in the intestinal tract. **(A)** The chemotaxis index of wild-type N2 worms trained on OP50 or *S.* Paratyphi A (mean ± SD, ** *p <*0.01, Student’s *t*-test). **(B)** After 12 h, 24 h and 48 h of *S.* Paratyphi A infection, pharyngeal pumping assay were performed (mean ± SD, *** *p* < 0.001, Student’s *t*-test). **(C)** The number of OP50 or *S.* Paratyphi A colonies/worm present in the intestine over 12 h (mean ± SD, *** *p <*0.001), 24h. (mean ± SD, *** *p <*0.001) and 48 h (mean ± SD, *** *p* < 0.001) treatment (n=10 in triplicates Student’s *t*-test). **(D)** Fluorescence images of representative worms raised on *E coli*::eGFP or *S*. Paratyphi A::eGFP (both carry the same eGFP expression plasmid). i Wild-type N2 worms fed E coli::eGFP for 24 h. ii N2 worms fasted for 24 hours after feeding *E coli*::eGFP. iii Wild-type N2 worms fed *S*. Paratyphi A::eGFP for 24h. iv N2 worms fasted for 24 hours after feeding *S*. Paratyphi A::eGFP. **(E)** Representative survival curves (*p* value by log-rank test) and graphs of mean lifespan of at least 3 independent experiments (mean ± SD, ** *p* < 0.01 and *** *p* < 0.001, NS represents not significant, Student’s *t*-test) of wild-type N2 worms on NGM plates containing untreated bacteria and heat-killed OP50 or *S.* Paratyphi A. **(F)** Representative survival curves (*p* value by log-rank test) and graphs of mean lifespan of at least 3 independent experiments (mean ± SD, * *p* < 0.05 and ** *p* < 0.01, NS represents not significant, Student’s *t*-test) of wild-type N2 worms on NGM plates containing untreated bacteria and OP50 or *S.* Paratyphi A secretions.

*C. elegans* possess the capacity to perceive, recognize and escape pathogenic bacteria. Under laboratory conditions, the chemotaxis behavior of worms implied their food preference. Our results showed that worms maintained with *E. coli* OP50 did not exhibit a bias to *E. coli* OP50 or *S.* Paratyphi A (chemotaxis index=0.182 ± 0.055). However, nematodes trained on *S.* Paratyphi A for 12 h strongly preferred OP50 relative to pathogenic *S.* Paratyphi A (chemotaxis index=0.677 ± 0.051, *p*=0.003203, **Figure 2A**; **Supplementary Table 6**). Pharyngeal pumping can reflect eating status. In general, when *C. elegans* were infected by pathogenic bacteria, they can reduce the bacteria intake to re-establish its behavior (40), therefore, we detected the number of pharyngeal pumps in 20s of nematode treated with OP50 or *S.* Paratyphi A. Our results showed that *S.* Paratyphi A exposure slowed the worms’ pharyngeal pumping at 12 h (OP50: 43.9 ± 3.1, *S.* Paratyphi A: 24.6 ± 2.9, *p*<0.0001), 24 h (OP50: 46.3 ± 4.1, *S.* Paratyphi A: 27.5 ± 3.2, *p*<0.0001) or 48 h (OP50: 50.7 ± 4.5, *S.* Paratyphi A: 35.4 ± 2.7, *p*<0.0001, **Figure 2B**; **Supplementary Table 7**). In general, *C. elegans* can respond to bacteria in the intestine and generate immune response to control the bacterial proliferation inside the body. Therefore, we also investigated whether *S.* Paratyphi A showed uncontrolled proliferation in the intestinal tract. Our results showed that the nematodes fed with *S.* Paratyphi A possessed more bacterial number than those fed *E. coli* OP50 for 12 h (OP50: 132 ± 37 CFU/worm, *S.* Paratyphi A: 2056 ± 534 CFU/worm, *p*<0.0001), 24 h (OP50: 1066 ± 196 CFU/worm, *S.* Paratyphi A: 11622 ± 1029 CFU/worm, *p*<0.0001) and 48 h (OP50: 3446 ± 316 CFU/worm, *S.* Paratyphi A: 379444 ± 28884 CFU/worm, *p*<0.0001, **Figure 2C**; **Supplementary Table 8**). Fluorescence images of representative animals starved for 24 hours after feeding *E. coli*::eGFP or *S*. Paratyphi A::eGFP further verified that *S*. Paratyphi A could proliferate in the intestinal lumen. After 24 hours of starvation, we observed evident fluorescence in *S*. Paratyphi A::eGFP-fasted animals but not in *E. coli*::eGFP-maintained or fasted worms (**Figure 2D**). We also discovered that the lifespan of worms showed no statistically significant differences between heat-killed *S*. Paratyphi A and OP50 bacteria (**Figure 2E**; **Supplementary Table 9**), and the lifespan of *S*. Paratyphi A secretions incubated with OP50 bacteria was similar to OP50 secretions (**Figure 2F**; **Supplementary Table 10**). The hazard ratios for heat-killed *S*. Paratyphi A and the secretions of living *S*. Paratyphi A were both reduced significantly. These results implied that the pathogenicity of *S*. Paratyphi A depends on living bacteria and is not mediated by secreting toxins.○

### *S*. Paratyphi A-induced immune response in *C. elegans* requires activation of the MAPK pathway

**Figure 3:**
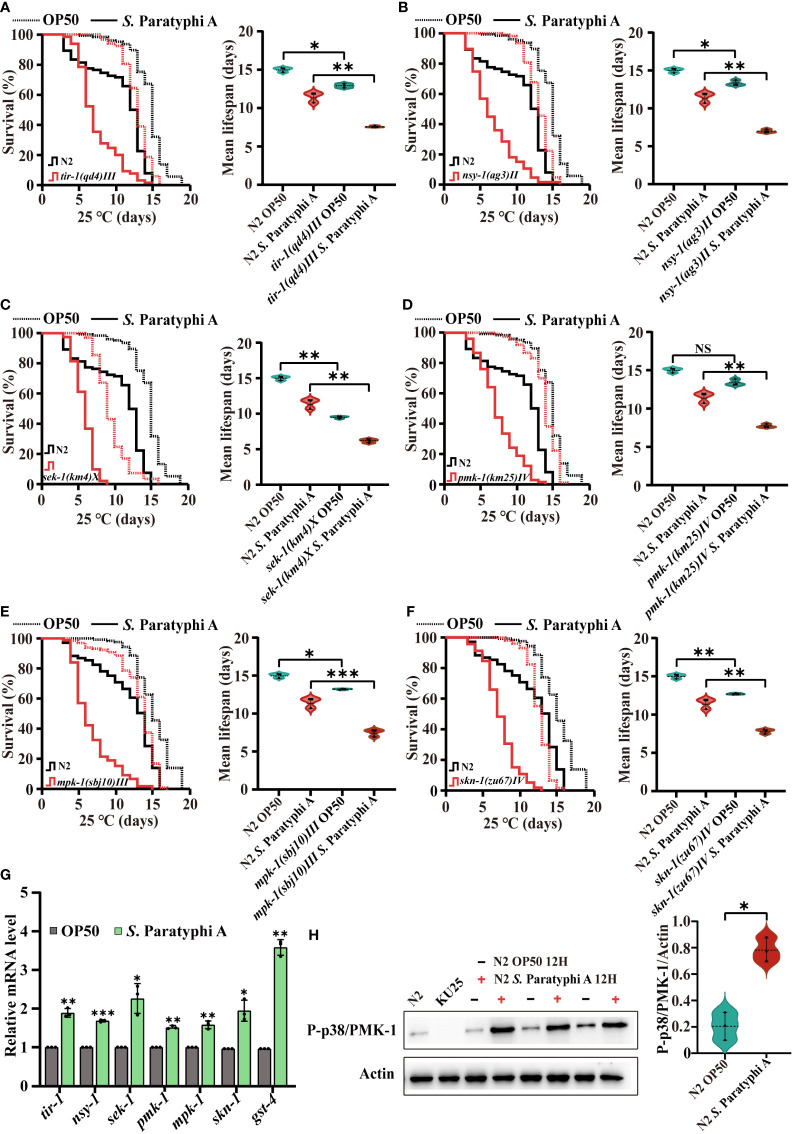
*S*. Paratyphi A-induced immune response in *C. elegans* requires activation of the MAPK pathway. Representative survival curves (*p* value by log-rank test) and graphs of mean lifespan of at least 3 independent experiments (mean ± SD, * *p* < 0.05, ** *p* < 0.01, *** *p* < 0.001, NS represents not significant, Student’s *t*-test) of wild-type N2 worms and mutants of *tir-1(qd4) III* **(A)** *, nsy-1(ag3) II* **(B)** *, sek-1(km4) X* **(C)** *, pmk-1(km25) IV* **(D)** *, mpk-1(sbj10) III* **(E)**, *skn-1(zu67) IV* **(F)** on NGM plates containing OP50 or *S.* Paratyphi A. **(G)** Relative mRNA levels of *tir-1*, *nsy-1*, *sek-1*, *pmk-1*, *mpk-1*, and *skn-1* were detected by quantitative RT-PCR. Statistical analyses were calculated using two-tailed Student’s *t* test; * represents *p* < 0.05, ** represents *p* < 0.01, and *** represents *p* < 0.001. **(H)** Western blot analyses and quantify the level of P-p38 MAPK/PMK-1 in N2 worms treated OP50 or *S.* Paratyphi A (KU25 *pmk-1(km25) IV* was analyzed to verify mutant). Actin was displayed as the loading control (* *p* < 0.05, two-tailed Student’s *t*-test).

To distinguish the signals involved in the interaction between *C. elegans* and *S*. Paratyphi A, we analyzed the lifespan of wild-type N2 and MAPK pathway mutants under exposure to *S*. Paratyphi A. The results showed that the mutants of *tir-1*, *nsy-1*, *sek-1* and *pmk-1* in the p38 MAPK pathway were more sensitive to *S*. Paratyphi A exposure than the wild type (**Figures 3A–D**, statistical information for all survival plots is presented in **Supplementary Table 11**). The *mpk-1* mutant in the ERK MAPK pathway also displayed greater sensitivity to *S*. Paratyphi A (**Figure 3E**; **Supplementary Table 11**). The results revealed that MAPK pathways function in the defense response of *C. elegans* to *S*. Paratyphi A infection. Reports have shown that the p38 MAPK pathway and *mpk-1* contribute to increase the nuclear localization of the transcription factor SKN-1 in the immune response (41, 42), therefore, we also detected the lifespan of wild-type N2 and the *skn-1(zu67)IV* mutant. We found that the lifespan of the *skn-1(zu67)IV* mutant was significantly decreased when nematodes were exposed to *S*. Paratyphi A, suggesting that *S*. Paratyphi A may activate the MAPK pathways in a SKN-1-dependent manner (**Figure 3F**; **Supplementary Table 11**). The hazard ratio for *tir-1*, *nsy-1*, *sek-1*, *pmk-1*, *mpk-1* and *skn-1* mutants exposed to *S*. Paratyphi A were higher than fed OP50 for these genes (**Supplementary Table 11**). We further confirmed the results by using quantitative RT-PCR to test the relative gene expression levels of *tir-1*, *nsy-1*, *sek-1*, *pmk-1, mpk-1*, and *skn-1* and the downstream target genes of SKN-1 (43) in the presence of *S*. Paratyphi A. The results showed that all these genes were upregulated when nematodes were exposed to *S*. Paratyphi A (**Figure 3G**; **Supplementary Table 12**). Considering activation of the p38 MAPK signaling pathway requires phosphorylation of p38 MAPK/PMK-1, we also compared the level of phosphorylated PMK-1 between *S*. Paratyphi A infection and OP50, the results displayed that *S*. Paratyphi A exposure has a higher level of activated P-p38/PMK-1 (**Figure 3H**), which further confirmed that MAPK pathways participated in the immune response of *C. elegans* to *S*. Paratyphi A exposure.

### Defense against *S*. paratyphi A may require the participation of DAF-2 and DAF-16

**Figure 4:**
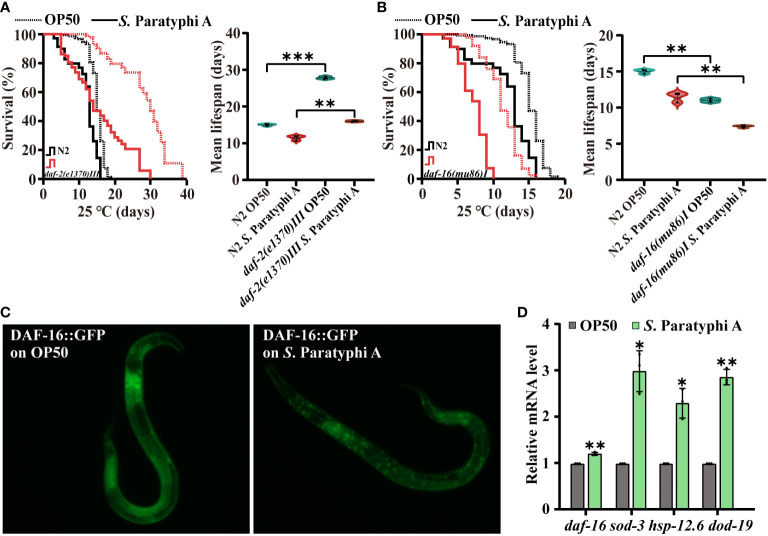
Defense against *S*. Paratyphi A may require the participation of DAF-2 and DAF-16. Representative survival curves (*p* value by log-rank test) and graphs of mean lifespan of at least 3 independent experiments (mean ± SD, ** *p* < 0.01 and *** *p* < 0.001, Student’s *t*-test) of wild-type N2 worms and mutants of *daf-2(e1370) III* **(A)** and *daf-16(mu86)I* **(B)**. **(C)** Fluorescence images of TJ356 zIs356 [*daf-16p*::*daf-16a/b*::*GFP* +*rol-6(su1006)*] grown on OP50 or *S*. Paratyphi A for 30 min. **(D)** Relative mRNA levels of *daf-16*, *sod-3*, *hsp-12.6* and *dod-19* were analyzed by quantitative RT–PCR (* *p* < 0.05 and ** *p* < 0.01, two-tailed Student’s *t*-test).

Inhibited IIS could enhance immunity of *C. elegans.* We detected the lifespan of wild-type N2 and the mutant of *daf-2(e1370) III* under *S*. Paratyphi A infection, and the results indicated that the mean lifespan of *daf-2(e1370) III* did increase compared to that of the wild-type N2 after *S*. Paratyphi A treatment (**Figure 4A**; **Supplemental Table 11**). We also found a higher hazard ratio for *daf-2* mutant fed *S*. Paratyphi A than that for OP50 (**Supplementary Table 11**). The immune response of worms may require the regulation of *daf-2*. The DAF-2 downstream molecular DAF-16, which is homolog of FOXO in mammal, servers as a key node in immunity according to reports (25). We sought to determine whether DAF-16 was involved in *S*. Paratyphi A infection and found that the mutant of *daf-16* enhanced the susceptibility of worms to *S*. Paratyphi A (**Figure 4B**; **Supplementary Table 11**). The hazard ratio for *daf-16* mutant treated *S*. Paratyphi A was higher than that for OP50 (**Supplementary Table 11**). In addition, the results from transgenic strain TJ356 zIs356 [*daf-16p*::*daf-16a/b*::*GFP +rol-6(su1006)*] indicated that *S*. Paratyphi A could significantly increase the nuclear localization of DAF-16 (**Figure 4C**; **Supplementary Figure 2**). We also analyzed the expression of *daf-16* and its downstream target genes *sod-3*, *hsp-12.6* and *dod-19* (44). The results indicated that *S*. Paratyphi A significantly upregulated the expression of *sod-3*, *hsp-12.6* and *dod-19* (**Figure 4D**; **Supplementary Table 12**). However, the level of *daf-16* didn^’^t show great change. *S*. Paratyphi A may enhance the activity of DAF-16, instead of increasing the level of expression. These results suggested that DAF-16 was activated in worms to defend against *S*. Paratyphi A.

### *S.* Paratyphi A may induce oxidative stress in *C. elegans*

**Figure 5:**
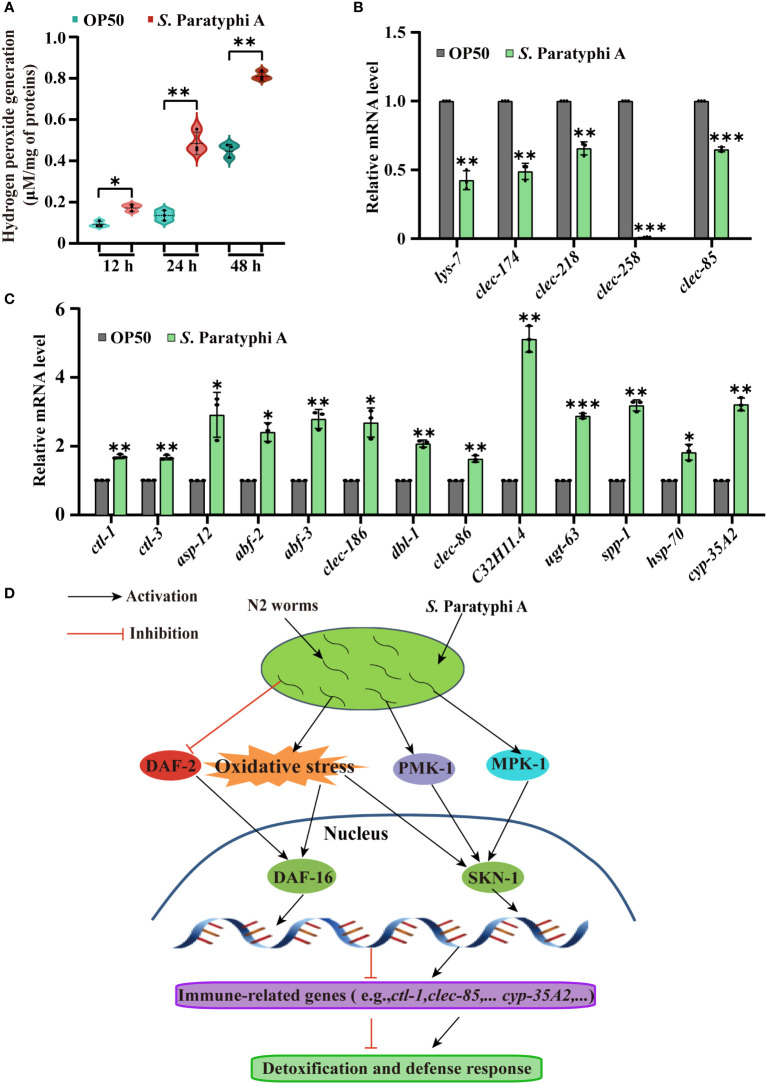
Stress response changes in *C. elegans* exposed to *S*. Paratyphi A. **(A)** Hydrogen peroxide assay after treatment with OP50 or *S*. Paratyphi A for 12, 24 or 48 hours (mean ± SD, * *p* < 0.05 and ** *p* < 0.01, Student’s *t*-test). **(B)** Relative mRNA levels of stress response-related genes lys-7, clec-174, clec-218, clec-258, clec-85 and (C) ctl-1, ctl-3, asp-12, abf-2, abf-3, clec-186, dbl-1, clec-86, C32H11.4, ugt-63, spp-1, hsp-70 and cyp-35A2 were analyzed by quantitative RT–PCR (* *p* < 0.05, ** *p* < 0.01, *** *p* < 0.001, two-tailed Student’s *t*-test). **(D)** Model of the interaction between *S*. Paratyphi A and *C. elegans.*

Pathogenic bacteria can induce oxidative stress in worms (45), and oxidative stress could defend worms against *S*. Paratyphi A infection but also inevitably lead to tissue damage of host (46). Hydrogen peroxide, the familiar reactive oxygen molecules in worms, can destroy the function of cells directly or indirectly. As reported, pathogenic bacteria, such as *Streptococcus pyogenes*, could produce millimolar concentrations of hydrogen peroxide to infect and kill nematodes (47, 48). To determine the interaction mechanism between *C. elegans* and *S.* Paratyphi A, we performed hydrogen peroxide and quantitative RT-PCR assay. We found that the level of hydrogen peroxide after treatment with *S*. Paratyphi A for 12, 24 or 48 hours was significantly increased (**Figure 5A**; **Supplementary Table 14**). Quantitative RT–PCR assays also showed increasing expression of *ctl-1* and *ctl-3*, both involved in hydrogen peroxide catabolic process under *S.* Paratyphi A infection (**Figure 5C**; **Supplementary Table 12**), suggesting that *S.* Paratyphi A induced the production of hydrogen peroxide and increased oxidative stress. To further confirm that *S.* Paratyphi A induced a stress response in worms, we detected the expression changes of stress response-related genes. We found that *asp-12*, *abf-2*, *abf-3*, *clec-186*, *dbl-1*, *clec-86*, *C32H11.4*, *ugt-63*, *spp-1*, *hsp-70* and *cyp-35A2* were upregulated when exposed to *S*. Paratyphi A (**Figure 5C**; **Supplementary Table 12**), while other stress response*-*related genes, including *lys-7*, *clec-174*, *clec-218*, *clec-258* and *clec-85* were downregulated (**Figure 5B**; **Supplementary Table 12**).

## Discussion

*S.* Paratyphi A shortened the lifespan of *C. elegans* hermaphrodites maintained at 25°C and impaired the thermotolerance at 35°C. The maximal effect of infection appeared at 35°C, indicating that 35°C is an optimal temperature for *S.* Paratyphi A to attack organisms. 37°C is the optimal temperature for *S.* Paratyphi A grown, the virulence of the bacteria may almost rise to the highest level at 35°C. *S.* Paratyphi A infection causes phenotypical changes such as arresting pharynx and accelerating the decline in body movement. We observed that worms grown on *S.* Paratyphi A lawn showed a greater hesitation toward food intake, which led to slower pharyngeal pumping and body movement. Reproduction is one of the best indicators for organism fitness, *C. elegans* infected with *S*. Paratyphi A showed fewer eggs which suggest that *S*. Paratyphi A may affect reproductive capacity. In general, worms did not show a simultaneous preference for OP50 and *S*. Paratyphi A. However, *S*. Paratyphi A-trained worms presented strong preference to OP50 in the food choice experiment, which made it clear that worms had identified the perniciousness of *S*. Paratyphi A with training. The CFU assay indicated that *S*. Paratyphi A colonized the intestinal region of *C. elegans*, and the fluorescence intensity in the intestine lumen further confirmed that *S*. Paratyphi A infect *C. elegans* through rapid proliferation in the intestinal tract. *S*. Paratyphi A colonizes the intestine and may have an effect on the locomotion and pharyngeal pumping rates of *C. elegans*. Our results confirmed that *S*. Paratyphi A can infect *C. elegans* and has an effect on phenotypic changes in worms. As the lifespan of secretions and heat-killed treatment of *S*. Paratyphi A showed no significant different from that of OP50 bacteria, the pathogenic bacteria mainly infect through colonizing the intestinal region.

MAPK is a conserved pathway in *C. elegans* innate immunity and is essential for defensing worms against pathogens such as *Pseudomonas aeruginosa*, *Cutibacterium acnes*, *Yersinia pestis*, and opportunistic *Proteus* species (49–52). In this study, the p38 MAPK and ERK MAPK pathway mutants showed greater sensitivity to *S*. Paratyphi A exposure than the wild type and higher hazard ratio to OP50, indicating that MAPK pathways may be activated in the immune response of *C. elegans* to *S*. Paratyphi A infection, and the higher level of phosphorylated PMK-1 in *S*. Paratyphi A infection obtained by western blot further confirmed the conclusion. The lifespan results of the *skn-1(zu67)IV* mutant verified that SKN-1 was involved in the defense response of *C. elegans* and may be activated by MAPK or oxidative stress, which will be further verified. These results were also obtained by quantitative RT-PCR. The long-lived *daf-2(e1370) III* showed a lifespan longer to that of the wild type under *S*. Paratyphi A exposure, and the expression of *daf-2* in *S*. Paratyphi A infect displayed slightly downregulated (**Supplementary Table 12**) indicated that inhibition of insulin signaling may defend worms against *S*. Paratyphi A infection. However, we also detected a higher hazard ratio for *daf-2* mutant in *S*. Paratyphi A, as *daf-2(e1370) III* is partially defective and extremely longevity, the effect of *daf-2* mutant fed *S*. Paratyphi A may ascribe complex signal regulation or other factors produced by *S*. Paratyphi A, which needs further exploration. The transcription factor DAF-16 of *C. elegans* integrate upstream signals to regulate the transcription of many genes related to stress, metabolism, and immunity (25). As reported, DAF-16 could be activated by *Bacillus thuringiensis*, *Pseudomonas aeruginosa*, *Cryptococcus neoformans* and *Cryptococcus gattii* infections (53–55). Our results found that the mutant *daf-16(mu86)I* enhanced susceptibility to *S*. Paratyphi A infection in *C. elegans* and the nuclear localization of DAF-16::GFP were enhanced in *S*. Paratyphi A exposure. All these results verify that worms activate DAF-16 to defend against *S*. Paratyphi A, and the increase in the expression of *sod-3*, *hsp-12.6* and *dod-19* confirmed this speculation. DAF-16 can be regulated by a variety of upstream signals, such as the typical IIS (56) and oxidative stress (25). Because *S*. Paratyphi A may regulate insulin signaling and stress response, DAF-16 may be activated by these signals (**Figure 5D**).

Oxidative stress also plays a crucial role in pathogen infection. The increase in the level of hydrogen peroxide after exposure to *S*. Paratyphi A confirmed that the oxidative stress response was involved in the interaction between *S*. Paratyphi A and *C. elegans*. The quantitative RT-PCR data indicated that the interaction between *S*. Paratyphi A and *C. elegans* was particularly enriched in the stress response. *C. elegans* upregulated genes related to the immune response to attack bacteria, while *S*. Paratyphi A downregulated certain detoxification genes so that they could colonize the worms.

In this study, we found that *S*. Paratyphi A could shorten the lifespan of *C. elegans* and cause phenotypical changes mainly by colonizing the intestinal region and regulating the stress response. MAPK and DAF-16 played a central role in *C. elegans* defense against *S*. Paratyphi A. Our findings revealed the interaction between *S*. Paratyphi A and *C. elegans* and provided insights for the development of novel pharmaceutical agents targeting bacterial infectious diseases.

## Data availability statement

The raw data supporting the conclusions of this article will be made available by the authors, without undue reservation.

## Author contributions

W-KZ and A-JD conceived the study. A-JD, X-CL, YD, H-JM, S-DP did most of the experiments. W-MZ, JT, BC and J-BH contributed to part of materials. W-KZ and A-JD wrote the manuscript. All authors contributed to the article and approved the submitted version.
