# Supplementary material for: Salmonella enterica serovar Paratyphi A-induced immune response in Caenorhabditis elegans depends on MAPK pathways and DAF-16
Source: Front Immunol. 2023 Apr 12;14:1118003. doi: 10.3389/fimmu.2023.1118003 (PMC10132459; doi:10.3389/fimmu.2023.1118003)
Supplement: Supplementary file 1 [file DataSheet_1.docx]

**Supplementary Materials**

**Contents:**

**Fig S1. The plates performed with chemotaxis behavior of N2**

**Fig S2. Subcellular localization of DAF-16/FOXO assay for 12 hours at 25 °C**

**Table S1. The effect of *S.* Paratyphi A on lifespan of N2**

**Table S2. The effects of *S.* Paratyphi A and other *E. coli* strains on lifespan of N2 at 25 °C**

**Table S3. The effect of *S.* Paratyphi A on lifespan of *hsf-1(sy441) I* at 35 ℃**

**Table S4. The effect of *S.* Paratyphi A on fast body movement**

**Table S5. The effect of *S.* Paratyphi A on eggs laid and spawning cycle of *C. elegans***

**Table S6. The effect of *S.* Paratyphi A on the chemotaxis behavior of N2**

**Table S7. The effect of *S.* Paratyphi A on pharyngeal pumping**

**Table S8. CFU assay of OP50 or *S*. Paratyphi A present in the intestine**

**Table S9. The effect of heat-killed *S.* Paratyphi A on lifespan of N2**

**Table S10. The effect of *S.* Paratyphi A secretions on lifespan of N2**

**Table S11. The effect of *S.* Paratyphi A on lifespan of mutants**

**Table S12. The effect of *S.* Paratyphi A on mRNA expression**

**Table S13. Primer sequences of genes used in qRT-PCR**

**Table S14. The effect of *S.* Paratyphi A on the expression of H_2_O_2_**

**
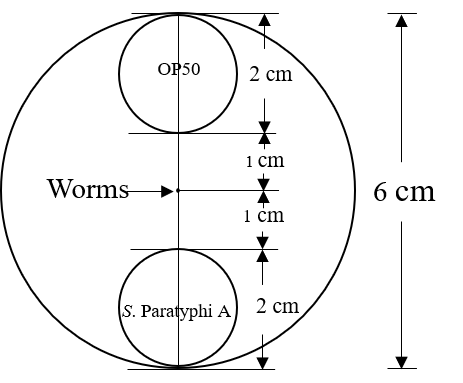
**

**Fig S1. The plates performed with chemotaxis behavior of N2**


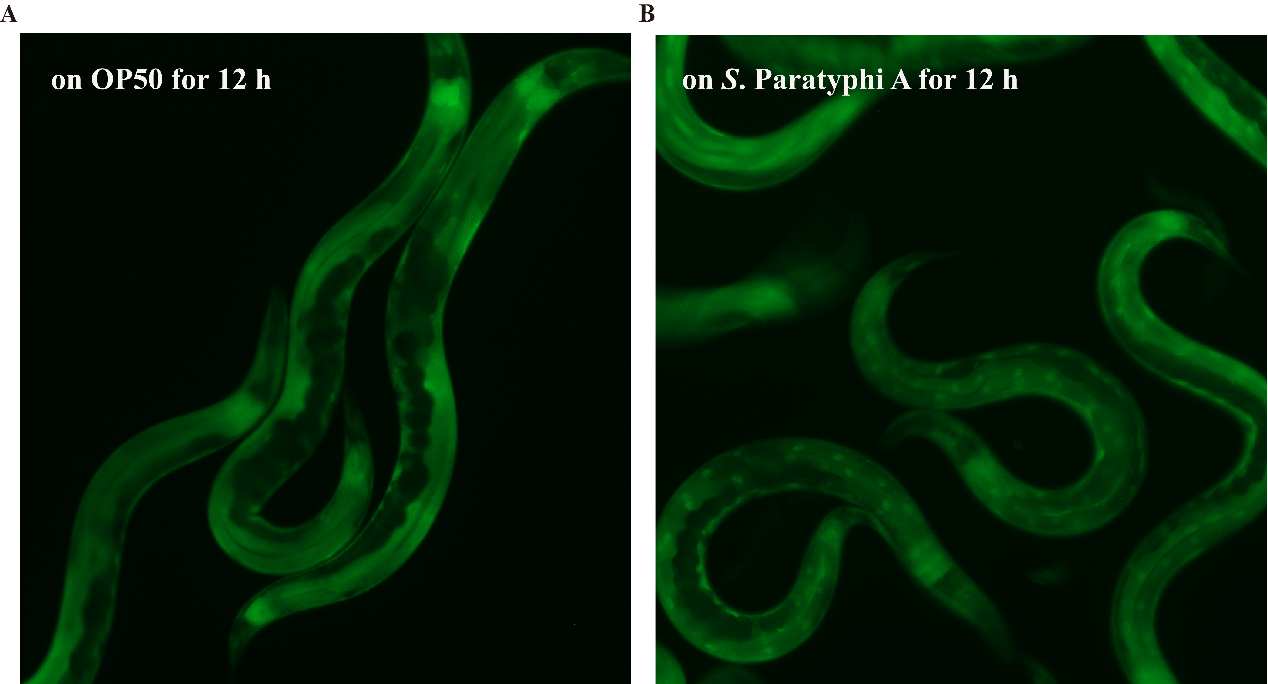


**Fig S2. Subcellular localization of DAF-16/FOXO assay for 12 hours at 25 °C**

Fluorescence images of TJ356 zIs356 [*daf-16p::daf-16a/b*::*GFP* +*rol-6(su1006)*] grown on OP50 (A) or *S*. Paratyphi A (B) for 12 h.

**Table S1. The effect of *S.* Paratyphi A on lifespan of N2**

| **Figure** | **Strains** | **Treatments** | **Mean lifespan**  **±SEM** | **% Change in mean lifespan** | ***P* value VS Control** | **N** |
| --- | --- | --- | --- | --- | --- | --- |
|  | **N2 (WT)** | |  |  |  |  |
| **1(A)** | EXP.1 | 25 °C / OP50 | 14.944±0.320 |  |  | 89 |
| **（days）** | EXP.1 | 25 °C / *S.* Paratyphi A | 11.010±0.237 | 26.3 | <0.0001 | 105 |
| **（days）** | EXP.2 | 25 °C / OP50 | 14.392±0.251 |  |  | 102 |
| **（days）** | EXP.2 | 25 °C / *S.* Paratyphi A | 11.000±0.427 | 23.6 | <0.0001 | 91 |
| **（days）** | EXP.3 | 25 °C / OP50 | 14.810±0.231 |  |  | 121 |
| **（days）** | EXP.3 | 25 °C / *S.* Paratyphi A | 10.932±0.273 | 26.2 | <0.0001 | 88 |
| For three independent experiments Student’s *t*-test 0.002117 | | | | |  |  |
| **1(B)** | EXP.1 | 35 °C / OP50 | 12.169±0.439 |  |  | 83 |
| **（Hours）** | EXP.1 | 35 °C / *S.* Paratyphi A | 8.247±0.303 | 32.2 | <0.0001 | 81 |
| **（Hours）** | EXP.2 | 35 °C / OP50 | 13.169±0.459 |  |  | 89 |
| **（Hours）** | EXP.2 | 35 °C / *S.* Paratyphi A | 9.284±0.334 | 29.5 | <0.0001 | 67 |
| **（Hours）** | EXP.3 | 35 °C / OP50 | 14.286±0.339 |  |  | 70 |
| **（Hours）** | EXP.3 | 35 °C / *S.* Paratyphi A | 9.724±0.274 | 31.9 | <0.0001 | 87 |
| For three independent experiments Student’s *t*-test 0.002829 | | | | |  |  |

Statistical analyses were carried out by Kaplan–Meier analysis, and log-rank test was applied to calculate *p* values. N: total number of nematodes tested.

**Table S2. The effects of *S.* Paratyphi A and other *E. coli* strains on lifespan of N2 at 25** °C

| **Strains** |  | **Mean lifespan**  **±SEM** | **% Change in**  **mean lifespan** | ***p* value VS**  ***S.* Paratyphi A** | **N** |
| --- | --- | --- | --- | --- | --- |
| ***S.* Paratyphi A** | EXP.1 | 10.356±0.277 |  |  | 101 |
|  | EXP.2 | 10.523±0.286 |  |  | 88 |
|  | EXP.3 | 10.569±0.239 |  |  | 109 |
| **OP50** | EXP.1 | 14.036±0.189 | 26.2 | <0.0001 | 139 |
|  | EXP.2 | 13.878±0.199 | 24.2 | <0.0001 | 139 |
|  | EXP.3 | 13.474±0.229 | 21.6 | <0.0001 | 95 |
| For three independent experiments Student’s *t*-test 0.004567 | | | | |  |
| **HT115** | EXP.1 | 13.834±0.219 | 25.1 | <0.0001 | 157 |
|  | EXP.2 | 13.677±0.297 | 23.1 | <0.0001 | 99 |
|  | EXP.3 | 13.645±0.260 | 22.5 | <0.0001 | 110 |
| For three independent experiments Student’s *t*-test 0.001443 | | | | |  |
| **JM109** | EXP.1 | 15.987±0.237 | 35.2 | <0.0001 | 149 |
|  | EXP.2 | 15.729±0.261 | 33.1 | <0.0001 | 140 |
|  | EXP.3 | 15.340±0.233 | 31.1 | <0.0001 | 141 |
| For three independent experiments Student’s *t*-test 0.002269 | | | | |  |

Statistical analyses were carried out by Kaplan–Meier analysis, and log-rank test was applied to calculate *p* values. N: total number of nematodes tested.

**Table S3. The effect of *S.* Paratyphi A on lifespan of** ***hsf-1(sy441) I* at 35 ℃**

N: total number of nematodes tested. Statistical analysis was based on Cox proportional hazards models.

| Genotype | *S*. Paratyphi A  Mean survival  time (hours) | SE for survival time (hours) | N | Hazard  Ratio | *p* value | *E. coli* OP50  Mean survival  time (hours) | SE for survival  time (hours) | N | Hazard  Ratio | *p* value |
| --- | --- | --- | --- | --- | --- | --- | --- | --- | --- | --- |
| Wild type | 9.651 | 0.352 | 63 |  |  | 13.585 | 0.420 | 82 |  |  |
|  | 9.831 | 0.291 | 71 |  |  | 14.150 | 0.381 | 80 |  |  |
|  | 9.367 | 0.300 | 79 |  |  | 13.543 | 0.415 | 70 |  |  |
|  |  |  |  |  |  |  |  |  |  |  |
| *hsf-1(sy441) I* | 5.257 | 0.158 | 105 | 7.332 | <0.0001 | 7.205 | 0.256 | 88 | 7.113 | <0.0001 |
|  | 5.275 | 0.178 | 102 | 7.080 | <0.0001 | 7.978 | 0.242 | 93 | 7.238 | <0.0001 |
|  | 5.554 | 0.175 | 112 | 5.260 | <0.0001 | 7.979 | 0.240 | 95 | 5.766 | <0.0001 |
| For three independent experiments Student’s *t*-test 0.552052 | | | | | 0.002799 | For three independent experiments Student’s *t*-test | | | | 0.001639 |

**Table S4. The effect of *S.* Paratyphi A on** **fast body movement**

| N2 | Treatment | Mean fast body movement time (days)±SEM | *p* value | N |
| --- | --- | --- | --- | --- |
| 1(C) EXP.1 | OP50 (5×10^9^ CFU/mL) | 9.514±0.143 |  | 105 |
| EXP.1 | OP50 (5×10^10^ CFU/mL) | 9.139±0.157 | 0.114 | 101 |
| EXP.1 | *S.* Paratyphi A (5×10^9^ CFU/mL) | 4.769±0.141 | <0.0001 | 108 |
| EXP.2 | OP50 (5×10^9^ CFU/mL) | 9.615±0.151 |  | 96 |
| EXP.2 | OP50 (5×10^10^ CFU/mL) | 9.539±0.165 | 0.831 | 89 |
| EXP.2 | *S.* Paratyphi A (5×10^9^ CFU/mL) | 4.822±0.160 | <0.0001 | 101 |
| EXP.3 | OP50 (5×10^9^ CFU/mL) | 9.505±0.158 |  | 101 |
| EXP.3 | OP50 (5×10^10^ CFU/mL) | 9.168±0.157 | 0.121 | 101 |
| EXP.3 | *S.* Paratyphi A (5×10^9^ CFU/mL) | 5.040±0.148 | <0.0001 | 101 |

Experiments were analyzed at 25 ℃. The log-rank (Kaplan-Meier) test was applied for statistical analysis. N: total number of nematodes tested.

**Table S5. The effect of *S.* Paratyphi A on** **eggs laid and** **spawning cycle of *C. elegans*.**

| **Figure 1 D, E**  **N2** | **Treatment** | **N** | | **Eggs laid** | **Spawning cycle** |  |
| --- | --- | --- | --- | --- | --- | --- |
| EXP.1 | 25 °C / OP50 | 15 | 183±21 | | 4.1±0.5 | |
| EXP.1 | 25 °C / *S*. Paratyphi A | 15 | 154±35 | | 3.1±0.4 | |
| EXP.2 | 25 °C / OP50 | 15 | 183±19 | | 3.9±0.5 | |
| EXP.2 | 25 °C / *S*. Paratyphi A | 15 | 157±20 | | 3.1±0.4 | |
| EXP.3 | 25 °C / OP50 | 15 | 185±17 | | 4.0±0.5 | |
| EXP.3 | 25 °C / *S*. Paratyphi A | 15 | 157±17 | | 3.3±0.5 | |
| ***p* value** |  |  | 2.634E-07 | | 1.022E-11 | |

N: total number of nematodes tested. *p* values were calculated by *t* test.

**Table S6. The effect of *S.* Paratyphi A on the** **chemotaxis behavior of N2**

| **Figure 2A**  **N2** | **Treatment** | **N** | **CI** |
| --- | --- | --- | --- |
| EXP.1 | 25 °C / OP50 | 66 | 0.242 |
| EXP.1 | 25 °C / *S*. Paratyphi A | 72 | 0.694 |
| EXP.2 | 25 °C / OP50 | 67 | 0.134 |
| EXP.2 | 25 °C / *S*. Paratyphi A | 79 | 0.620 |
| EXP.3 | 25 °C / OP50 | 65 | 0.169 |
| EXP.3 | 25 °C / *S*. Paratyphi A | 92 | 0.717 |
| ***p* value** |  |  | 0.003203 |

N: total number of nematodes tested. CI: chemotaxis index = No. of animals moved toward OP50 -No. of animals moved toward *S*. Paratyphi A /Total No. of worms assayed.

**Table S7. The effect of *S.* Paratyphi A on** **pharyngeal pumping**

| **Figure 2B**  **N2** | **Treatment** | **Number of pharyngeal pumping (per 20 seconds)**  **Mean ± SEM** | **N** | **Number of pharyngeal pumping (per 20 seconds)**  **Mean ± SEM** | **N** | **Number of pharyngeal pumping (per 20 seconds)**  **Mean ± SEM** | **N** |
| --- | --- | --- | --- | --- | --- | --- | --- |
|  |  | **12 h** | | **24 h** | | **48 h** | |
| EXP. 1 | 25 °C / OP50 | 44.3 ± 3.335 | 10 | 46.5 ± 5.017 | 10 | 55.5 ± 2.369 | 10 |
| EXP. 1 | 25 °C / *S*. Paratyphi A | 24.5 ± 2.915 | 10 | 26.3 ± 2.791 | 10 | 37.8 ± 1.687 | 10 |
| EXP. 2 | 25 °C / OP50 | 42.0 ± 2.539 | 10 | 47.3 ± 2.791 | 10 | 47.7 ± 3.433 | 10 |
| EXP. 2 | 25 °C / *S*. Paratyphi A | 24.3± 2.406 | 10 | 30.3 ± 2.214 | 10 | 34.0 ± 2.000 | 10 |
| EXP. 3 | 25 °C / OP50 | 45.3± 2.627 | 10 | 45.0 ± 4.216 | 10 | 49.0 ± 2.944 | 10 |
| EXP. 3 | 25 °C / *S*. Paratyphi A | 25.0± 3.651 | 10 | 26.0± 2.828 | 10 | 34.3± 2.497 | 10 |
| ***p* value** |  | 2.531E-21 |  | 2.041E-20 |  | 1.890E-18 |  |

N: total number of nematodes tested. Statistical analysis was based on *t* test.

| **Figure 2C**  **N2** | **Treatment** | **12 h**  **CFU/Worm** | **24 h CFU/Worm** | **48h CFU/Worm** | **N** |
| --- | --- | --- | --- | --- | --- |
| EXP. 1 | 25 °C / OP50 | 137 | 1080 | 3410 | 10 |
| EXP. 1 | 25 °C / *S*. Paratyphi A | 2033 | 11967 | 378000 | 10 |
| EXP. 2 | 25 °C / OP50 | 140 | 1050 | 3437 | 10 |
| EXP. 2 | 25 °C / *S*. Paratyphi A | 2067 | 11867 | 378667 | 10 |
| EXP. 3 | 25 °C / OP50 | 120 | 1067 | 3490 | 10 |
| EXP. 3 | 25 °C / *S*. Paratyphi A | 2067 | 11033 | 381667 | 10 |
| ***p* value** |  | 3.939E-06 | 1.261E-09 | 2.091E-10 |  |

**Table S8. CFU assay of OP50 or *S*. Paratyphi A present in the intestine**

N: total number of nematodes tested. Statistical analysis was based on *t* test.

| Figure 2E  N2 | *S*. Paratyphi A  Mean survival  time (days) | SE for survival time (days) | N | Hazard  Ratio | *p* value | *E. coli* OP50  Mean survival  time (days) | SE for survival  time (days) | N | Hazard  Ratio | *p* value |
| --- | --- | --- | --- | --- | --- | --- | --- | --- | --- | --- |
| Untreated | 11.031 | 0.313 | 97 |  |  | 15.113 | 0.245 | 115 |  |  |
|  | 11.095 | 0.310 | 95 |  |  | 14.504 | 0.238 | 117 |  |  |
|  | 11.564 | 0.325 | 94 |  |  | 14.435 | 0.238 | 115 |  |  |
|  |  |  |  |  |  |  |  |  |  |  |
| Heat-killed | 17.143 | 0.511 | 84 | 0.112 | <0.0001 | 17.010 | 0.342 | 100 | 0.501 | <0.0001 |
|  | 17.131 | 0.411 | 84 | 0.116 | <0.0001 | 16.917 | 0.328 | 108 | 0.389 | <0.0001 |
|  | 17.113 | 0.316 | 97 | 0.102 | <0.0001 | 16.522 | 0.332 | 113 | 0.480 | <0.0001 |
| For three independent experiments Student’s *t*-test | | | | 0.011183 | 0.000893 | For three independent experiments Student’s *t*-test | | | | 0.004956 |

**Table S9. The effect of heat-killed *S.* Paratyphi A on lifespan of N2**

N: total number of nematodes tested. Statistical analysis was based on Cox proportional hazards models.

| Figure 2F  N2 | *S*.Paratyphi A  Mean survival  time (days) | SE for survival time (days) | N | Hazard  Ratio | *p* value | *E.coli* OP50  Mean survival  time (days) | SE for survival  time (days) | N | Hazard  Ratio | *p* value |
| --- | --- | --- | --- | --- | --- | --- | --- | --- | --- | --- |
| Untreated | 9.045 | 0.177 | 66 |  |  | 14.360 | 0.218 | 114 |  |  |
|  | 9.239 | 0.151 | 71 |  |  | 13.861 | 0.287 | 101 |  |  |
|  | 9.360 | 0.178 | 75 |  |  | 13.887 | 0.214 | 124 |  |  |
|  |  |  |  |  |  |  |  |  |  |  |
| Secretions | 14.738 | 0.437 | 80 | 0.110 | <0.0001 | 14.329 | 0.486 | 76 | # | 0.167 |
|  | 14.500 | 0.462 | 74 | 0.122 | <0.0001 | 14.247 | 0.461 | 77 | # | 0.106 |
|  | 14.218 | 0.338 | 101 | 0.127 | <0.0001 | 13.773 | 0.468 | 66 | # | 0.566 |
| For three independent experiments Student’s *t*-test | | | | | 0.002086 | For three independent experiments Student’s *t*-test | | | | 0.655313 |

**Table S10. The effect of *S.* Paratyphi A** **secretions on lifespan of N2**

N: total number of nematodes tested. Statistical analysis was based on Cox proportional hazards models.

#: no calculate (because *p* >0.05).

**Table S11. The effect of *S.* Paratyphi A on lifespan of mutants**

| *S.* Paratyphi A  Genotype | Mean survival  time (days) | SE for survival time (days) | No.of  nematodes tested | Hazard ratio | *p* value | OP50  Mean survival  time (days) | SE for survival time (days) | No.of nematodes tested | Hazard ratio | *p* value |
| --- | --- | --- | --- | --- | --- | --- | --- | --- | --- | --- |
| Wild type  3A-D EXP.1  3E-F EXP.2  4A-B EXP.3 | 10.725  11.870  11.899 | 0.394  0.479  0.458 | 102  69  69 |  |  | 14.688  15.226  15.134 | 0.200  0.218  0.174 | 125  124  149 |  |  |
| *tir-1(qd4) III*  3A EXP.1  EXP.2  EXP.3 | 7.523  7.708  7.551 | 0.340  0.376  0.304 | 65  65  89 | 2.589  3.524  3.561 | <0.0001  <0.0001  <0.0001 | 12.915  12.663  13.165 | 0.167  0.159  0.179 | 118  80  97 | 2.447  3.453  2.776 | <0.0001  <0.0001  <0.0001 |
| For three independent experiments Student’s *t*-test | | | | | 0.008171 | For three independent experiments Student’s *t*-test | | | | 0.012531 |
| *nsy-1(ag3) II*  3B EXP.1  EXP.2  EXP.3 | 6.821  6.898  7.241 | 0.371  0.282  0.335 | 67  98  87 | 2.983  3.937  3.884 | <0.0001  <0.0001  <0.0001 | 13.111  13.133  13.722 | 0.161  0.188  0.198 | 108  83  108 | 2.358  2.711  1.768 | <0.0001  <0.0001  <0.0001 |
| For three independent experiments Student’s *t*-test | | | | | 0.004898 | For three independent experiments Student’s *t*-test | | | | 0.014345 |
| *sek-1(km4) X* |  |  |  |  |  |  |  |  |  |  |
| 3C EXP.1 | 5.899 | 0.125 | 119 | 6.821 | <0.0001 | 9.587 | 0.215 | 104 | 5.450 | <0.0001 |
| EXP.2 | 6.217 | 0.131 | 115 | 8.772 | <0.0001 | 9.573 | 0.214 | 110 | 6.298 | <0.0001 |
| EXP.3 | 6.401 | 0.168 | 137 | 9.066 | <0.0001 | 9.298 | 0.234 | 104 | 6.232 | <0.0001 |
| For three independent experiments Student’s *t*-test | | | | | 0.002264 | For three independent experiments Student’s *t*-test | | | | 0.001592 |
| *S.* Paratyphi A  Genotype | **Mean survival**  **time (days)** | **SE for survival time (days)** | **No.of**  **nematodes tested** | **Hazard ratio** | ***p* value** | **OP50**  **Mean survival**  **time (days)** | **SE for survival time (days)** | **No.of nematodes tested** | **Hazard ratio** | ***p* value** |
| *pmk-1(km25) IV*  3D EXP.1  EXP.2  EXP.3 | 7.587  7.597  7.990 | 0.311  0.368  0.319 | 75  77  103 | 3.242  2.892  2.757 | <0.0001  <0.0001  <0.0001 | 13.850  13.198  13.120 | 0.187  0.200  0.233 | 113  116  117 | 1.506  2.344  2.020 | 0.002  <0.0001  <0.0001 |
| For three independent experiments Student’s *t*-test | | | | | 0.007771 | For three independent experiments Student’s *t*-test | | | | 0.054052 |
| *mpk-1(sbj10) III* |  |  |  |  |  |  |  |  |  |  |
| 3E EXP.1  EXP.2  EXP.3 | 6.951  7.762  7.770 | 0.318  0.363  0.375 | 82  63  61 | 2.638  3.372  3.626 | <0.0001  <0.0001  <0.0001 | 13.258  13.230  13.127 | 0.242  0.284  0.281 | 132  100  71 | 1.643  2.067  2.175 | <0.0001  <0.0001  <0.0001 |
| For three independent experiments Student’s *t*-test | | | | | 0.000824 | For three independent experiments Student’s *t*-test | | | | 0.010888 |
| *skn-1(zu67)IV* |  |  |  |  |  |  |  |  |  |  |
| 3F EXP.1  EXP.2  EXP.3 | 7.505  7.922  8.046 | 0.235  0.254  0.239 | 91  90  87 | 3.892  5.143  4.853 | <0.0001  <0.0001  <0.0001 | 12.641  12.697  12.798 | 0.148  0.178  0.181 | 103  99  89 | 3.407  3.308  3.657 | <0.0001  <0.0001  <0.0001 |
| For three independent experiments Student’s *t*-test | | | | | 0.003846 | For three independent experiments Student’s *t*-test | | | | 0.003675 |
| *daf-2(e1370) III* |  |  |  |  |  |  |  |  |  |  |
| 4A EXP.1 | 15.805 | 0.856 | 87 | 0.381 | <0.0001 | 28.205 | 0.820 | 83 | 0.077 | <0.0001 |
| EXP.2 | 15.989 | 0.724 | 89 | 0.398 | <0.0001 | 27.750 | 0.783 | 76 | 0.084 | <0.0001 |
| EXP.3 | 16.192 | 0.889 | 78 | 0.389 | <0.0001 | 27.495 | 0.804 | 91 | 0.090 | <0.0001 |
| For three independent experiments Student’s *t*-test | | | | | 0.004293 | For three independent experiments Student’s *t*-test | | | | 0.000795 |
| *daf-16(mu86)I* |  | | | |  |  |  |  |  |  |
| 4B EXP.1  EXP.2  EXP.3 | 7.203  7.455  7.495 | 0.224  0.180  0.194 | 69  88  99 | 5.325  6.496  7.946 | <0.0001  <0.0001  <0.0001 | 11.264  11.025  10.767 | 0.255  0.277  0.271 | 87  79  86 | 4.076  5.037  5.761 | <0.0001  <0.0001  <0.0001 |
| For three independent experiments Student’s *t*-test | | | | | 0.005133 | For three independent experiments Student’s *t*-test | | | | 0.005245 |

Statistical analysis was based on Cox proportional hazards models.

**Table S12. The effect of *S.* Paratyphi A on mRNA expression**

| **Figure** |  | ***tir-1*** | ***nsy-1*** | ***sek-1*** | ***pmk-1*** | ***mpk-1*** | ***skn-1*** | ***gst-4*** |  |  |  |  |
| --- | --- | --- | --- | --- | --- | --- | --- | --- | --- | --- | --- | --- |
| **3(G)** | EXP.1 | 2.020 | 1.707 | 2.577 | 1.590 | 1.669 | 1.896 | 3.766 |  |  |  |  |
|  | EXP.2 | 1.808 | 1.650 | 2.375 | 1.512 | 1.603 | 2.291 | 3.709 |  |  |  |  |
|  | EXP.3 | 1.845 | 1.700 | 1.834 | 1.503 | 1.474 | 1.765 | 3.388 |  |  |  |  |
|  | **Mean** | 1.891 | 1.686 | 2.262 | 1.535 | 1.582 | 1.984 | 3.621 |  |  |  |  |
|  | **SEM** | 0.113 | 0.031 | 0.384 | 0.047 | 0.099 | 0.274 | 0.204 |  |  |  |  |
| ***P* value** | | 0.0053 | 0.0007 | 0.0296 | 0.0026 | 0.0095 | 0.025 | 0.0020 |  |  |  |  |
|  |  |  |  |  |  |  |  |  |  |  |  |  |
| **Figure** |  | ***daf-16*** | ***sod-3*** | ***hsp-12.6*** | ***dod-19*** | ***daf-2*** |  |  |  |  |  |  |
| **4(D)** | EXP.1 | 1.203 | 3.376 | 2.616 | 2.754 | 0.912 |  |  |  |  |  |  |
|  | EXP.2 | 1.253 | 2.517 | 1.976 | 2.813 | 0.832 |  |  |  |  |  |  |
|  | EXP.3 | 1.195 | 3.134 | 2.352 | 3.071 | 0.884 |  |  |  |  |  |  |
|  | **Mean** | 1.217 | 3.010 | 2.315 | 2.879 | 0.876 |  |  |  |  |  |  |
|  | **SEM** | 0.032 | 0.443 | 0.321 | 0.168 | 0.041 |  |  |  |  |  |  |
| ***P* value** | | 0.0069 | 0.0158 | 0.0193 | 0.0027 | 0.0342 |  |  |  |  |  |  |
|  |  | | | |  |  |  |  |  |  |  |  |
| **Figure** |  | ***lys-7*** | ***clec-174*** | ***clec-218*** | ***clec-258*** | ***clec-85*** |  |  |  |  |  |  |
| **5(B)** | EXP.1 | 0.366 | 0.523 | 0.681 | 0.009 | 0.649 |  |  |  |  |  |  |
|  | EXP.2 | 0.410 | 0.421 | 0.602 | 0.011 | 0.667 |  |  |  |  |  |  |
|  | EXP.3 | 0.500 | 0.524 | 0.688 | 0.014 | 0.633 |  |  |  |  |  |  |
|  | **Mean** | 0.425 | 0.489 | 0.657 | 0.011 | 0.650 |  |  |  |  |  |  |
|  | **SEM** | 0.068 | 0.059 | 0.048 | 0.002 | 0.017 |  |  |  |  |  |  |
| ***P* value** | | 0.0047 | 0.0044 | 0.0064 | <0.0001 | 0.0008 |  |  |  |  |  |  |
|  | | |  | |  |  |  |  |  | | | |
| **Figure**  **5(C)** | | | ***asp-12 abf-2*** | | ***abf-3*** | ***clec-186*** | ***dbl-1*** | ***clec-86*** | ***C32H11.4*** | | | |
| EXP.1 | | 3.203 | 2.452 | 2.935 | 2.215 | 2.154 | 1.550 | 5.500 | | |  |  |
| EXP.2 | | 3.370 | 2.123 | 2.474 | 3.028 | 2.119 | 1.610 | 5.105 | | |  |  |
| EXP.3 | | 2.169 | 2.659 | 2.972 | 2.821 | 1.960 | 1.735 | 4.746 | | |  |  |
| **Mean** | | 2.914 | 2.411 | 2.794 | 2.688 | 2.078 | 1.632 | 5.117 | | |  |  |
| **SEM** | | 0.651 | 0.270 | 0.277 | 0.423 | 0.103 | 0.094 | 0.377 | | |  |  |
| ***P* value** | | 0.0365 | 0.0120 | 0.0079 | 0.0203 | 0.0030 | 0.0074 | 0.0028 | | |  |  |
|  | |  |  |  |  |  |  |  | | |  |  |
|  | | ***ugt-63*** | ***spp-1*** | ***hsp-70*** | ***cyp-35A2*** | ***ctl-1*** | ***ctl-3*** |  | | |  |  |
| EXP.1 | | 2.807 | 3.002 | 1.855 | 3.192 | 1.671 | 1.618 |  | | |  |  |
| EXP.2 | | 2.955 | 3.284 | 2.033 | 3.415 | 1.647 | 1.742 |  | | |  |  |
| EXP.3 | | 2.880 | 3.278 | 1.584 | 3.048 | 1.764 | 1.638 |  | | |  |  |
| **Mean** | | 2.881 | 3.188 | 1.824 | 3.218 | 1.694 | 1.666 |  | | |  |  |
| **SEM** | | 0.074 | 0.161 | 0.226 | 0.184 | 0.062 | 0.066 |  | | |  |  |
| ***P* value** | | 0.0005 | 0.0018 | 0.0242 | 0.0023 | 0.0026 | 0.0033 |  | | |  |  |

Every value of number representatives the expression level of genes and was calculated by 2^–△△CT^ method and *cdc-42* was applied to be the reference genes. Two-tailed *t* test was used to analyze *p* values.

**Table S13. Primer sequences of genes used in qRT-PCR**

| **Gene** | **Type** | **Sequence** |
| --- | --- | --- |
| ***cdc-42*** | F | 5’- TGTCGGTAAAACTTGTCTCCTG -3’ |
|  | R | 5’- ATCCTAATGTGTATGGCTCGC-3’ |
| ***tir-1*** | F | 5’-TTGGGTGCACAAAGAGCTGA-3’ |
|  | R | 5’-GGTCGGTGTCGTTCTGTTCA-3’ |
| ***nsy-1*** | F | 5’-AGCGGCTCGATCAACAAGAA-3’ |
|  | R | 5’-CCCATTCCACCGATATGCGA-3’ |
| ***sek-1*** | F | 5’-CACTGTTTGGCGACGATGAG-3’ |
|  | R | 5’-ATTCCGTCCACGTTGCTGAT-3’ |
| ***pmk-1*** | F | 5’-CCAAAAATGACTCGCCGTGA-3’ |
|  | R | 5’-CTTTTGCAGTTGGACGACGA-3’ |
| ***mpk-1*** | F | 5’-GCTCCGGGATCATTGGCATA-3’ |
|  | R | 5’-CTTCTCCGTCGGCCATCTTT-3’ |
| ***skn-1*** | F | 5’-CTGGCATCCTCTACCACCAC-3’ |
|  | R | 5’- TTGGTGATGATGGCCGTGTT -3’ |
| ***gst-4*** | F | 5’- TCCGTCAATTCACTTCTTCCG -3’ |
|  | R | 5’- AAGAAATCATCACGGGCTGG -3’ |
| ***daf-16*** | F | 5’-TCGTCTCGTGTTTCTCCAGC-3’ |
|  | R | 5’-TAATCGGCTTCGACTCCTGC-3’ |
| ***sod-3*** | F | 5’-AGCATCATGCCACCTACGTGA-3’ |
|  | R | 5’-CACCACCATTGAATTTCAGCG-3’ |
| ***hsp-12.6*** | F | 5’- GTGATGGCTGACGAAGGAAC -3’ |
|  | R | 5’- GGGAGGAAGTTATGGGCTTC -3’ |
| ***dod-19*** | F | 5’- ACCGTTCCCAGTTTTACAGTCC -3’ |
|  | R | 5’- TATTTTGAGGCGCGGATACAC-3’ |
| ***lys-7*** | F | 5’- GTACAGCGGTGGAGTCACTG -3’ |
|  | R | 5’- GCCTTGAGCACATTTCCAGC-3’ |
| ***clec-174*** | F | 5'-CGATGATGCTCATAACTGGTG-3' |
|  | R | 5'-CCCATTCCAAGAGACCAGAA-3' |
| ***clec-218*** | F | 5'-ACGCCGGAGATTATGTGGAT-3' |
|  | R | 5'-CCAACTCCTTGAGCCATTTCAG-3' |
| ***clec-258*** | F | 5'-CAGCTGGGCCTTCATGGCTA-3' |
|  | R | 5'-CCGGCGTAGACGGCCAAA-3' |
| ***clec-85*** | F | 5'-CCAATGGGATGACGGAACCA-3' |
|  | R | 5'-CTTCTGTCCAGCCAACGTCT-3' |
| ***asp-12*** | F | 5'-CGGAGATGGATGACATTTGAG-3’ |
|  | R | 5'-ACCACAGTTGCCGAGCAC-3’ |
| ***abf-2*** | F | 5'-CCGTTCCCTTTTCCTTGCAC-3’ |
|  | R | 5'-GACGACCGCTTCGTTTCTTG-3’ |
| ***abf-3*** | F | 5'-AACAGATTGGGGTCAGCTCG-3’ |
|  | R | 5'-TGGAGACCATTATTGCCGGG-3’ |
| ***clec-186*** | F | 5'-GTGGAAAATGGCAATGGTTT -3’ |
|  | R | 5'-AACAACTCCCTCGTTTGGTG-3’ |
| ***dbl-1*** | F | 5'-TTTTGCGGCGAACAAATCGT-3’ |
|  | R | 5'-TTCGCTGTTGCCTGTTTGTG-3’ |
| ***clec-86*** | F | 5'-TCGAAAACAACTGGATTCAAAAACT-3' |
|  | R | 5'-CGCTCCCATCTGTCCATCTC-3' |
| ***C32H11.4*** | F | 5'-ACCGAGCCAGGAGGTTATCT-3’ |
|  | R | 5'-TCCCGATGTTGATTTTGACC-3’ |
| ***ugt-63*** | F | 5'-TGCTCATTTTCGCCAGGACA-3’ |
|  | R | 5'-CGTAGGCGACTGCCTGATG-3’ |
| ***spp-1*** | F | 5'-TGGACTATGCTGTTGCCGTT-3’ |
| ***hsp-70***  ***cyp-35A2*** | R  F  R  F  R | 5'-ACGCCTTGTCTGGAGAATCC-3’  5'-AATGAACCAACTGCTGCTGCTCTT-3’  5'-TGTCCTTTCCGGTCTTCCTTTTG-3’  5'-TTCTGTGCTTTTGGGATACC-3’  5'-CATAATCCGCAATGCTCAGG-3’ |
| ***daf-2*** | F | 5'-TGAAAGCGAAGCAGCGAGAAGG-3’ |
|  | R | 5'-CGTCCGAACTTCCGCATCACTC-3’ |
| ***ctl-1*** | F | 5'-TCGTTCATGCCAAGGGAGC-3’ |
|  | R | 5'-GATTCTCCAGCGACCGTTGA-3’ |
| ***ctl-3*** | F | 5'-AATGCCAATGCTTCCCCACA-3’ |
|  | R | 5'-GCAGGTGGGGTTCCTGATT-3’ |

**Table S14. The effect of *S.* Paratyphi A on the expression of H_2_O_2_**

| **Figure 5A**  **N2** | **Treatment** | **12 h**  **(μM/mg of proteins)** | **24 h**  **(μM/mg of proteins)** | **48 h**  **(μM/mg of proteins)** |
| --- | --- | --- | --- | --- |
| EXP. 1 | 25 °C / OP50 | 0.085 | 0.111 | 0.416 |
| EXP. 1 | 25 °C / *S*. Paratyphi A | 0.156 | 0.454 | 0.837 |
| EXP. 2 | 25 °C / OP50 | 0.086 | 0.136 | 0.468 |
| EXP. 2 | 25 °C / *S*. Paratyphi A | 0.188 | 0.554 | 0.806 |
| EXP. 3 | 25 °C / OP50 | 0.110 | 0.160 | 0.477 |
| EXP. 3 | 25 °C / *S*. Paratyphi A | 0.178 | 0.464 | 0.796 |
| ***p* value** |  | 0.016518 | 0.008687 | 0.007434 |

Student *t* test was applied for statistical analysis.
